# Health-Related Quality of Life in Childhood and Adolescence: The Interrelation with Level of Adherence to Mediterranean Diet and Dietary Trajectories: Highlights from the DIATROFI Program (2021–2022)

**DOI:** 10.3390/nu15081829

**Published:** 2023-04-10

**Authors:** Dimitrios V. Diamantis, Konstantinos Katsas, Dimitrios Kalogiannis, Matina Kouvari, Athena Linos

**Affiliations:** 1 Institute of Preventive Medicine Environmental and Occupational Health PROLEPSIS, 15121 Athens, Greece; dimitrios.v.diamantis@gmail.com (D.V.D.); k.katsas@prolepsis.gr (K.K.);; 2 Medical School, National and Kapodistrian University of Athens, 11527 Athens, Greece; 3 Department of Nutrition and Dietetics, School of Health Science and Education, Harokopio University, 17676 Athens, Greece; 4 Discipline of Nutrition and Dietetics, Faculty of Health, University of Canberra, Canberra, ACT 2601, Australia; 5 Functional Foods and Nutrition Research (FFNR) Laboratory, University of Canberra, Bruce, ACT 2617, Australia

**Keywords:** quality of life, wellbeing, children, students, Mediterranean diet, DIATROFI program

## Abstract

Children’s dietary habits can have a key role in contributing to an improvement in their Health-Related Quality of Life (HRQoL). This study aims to assess the connection between Mediterranean diet adherence and HRQoL in a sample of Greek students, utilizing data from the DIATROFI program. The parents of 3774 students (mean age 7.8 (2.6) years) reported their children’s HRQoL and level of adherence to the Mediterranean dietary pattern at the beginning and end of the 2021–2022 school year. At baseline, most students’ adherence was characterized as moderate (55.2%) or high (25.1%). Students with moderate or high adherence tο the Mediterranean diet were less likely to report a total HRQoL below the median at baseline (OR = 0.56, 95%CI = 0.44, 0.70), along with all its dimensions (physical, emotional, social, and school functions). A one-unit improvement in KIDMED score _(beginning–end of schoolyear)_ was associated with the likelihood of an improvement in total HRQoL _(beginning–end of schoolyear)_ (OR = 1.09, 95%CI = 1.02, 1.17), emotional (OR = 1.09, 95%CI = 1.02, 1.17), and social functions (OR = 1.13, 95%CI = 1.05, 1.22), but not with physical and school functions. The health benefits of the Mediterranean diet in children may not be limited to disease prevention but also extend to their overall wellbeing.

## 1. Introduction

Ensuring the wellbeing of children and adolescents is a crucial step toward healthy development, healthy behaviors, and future achievements [1]. However, evaluating the level of wellbeing in childhood may seem challenging and require researchers to consider factors beyond their physiological health. They should approach it as a complex phenomenon, accounting for their mental and social wellbeing and other vital aspects from the child’s point of view. Therefore, most studies evaluate the quality of life, a term that describes the “individual’s perception of position in life in the context of the culture and value systems in which they live and concerning goals, expectations, standards, and concerns” [2]. Such a multidimensional concept in children is not well established in the literature, but a consensus, along with most quality-of-life measuring tools, accounts for the following aspects: physiological, psychological/mental, social, and school/cognitive performance [3].

Poor nutritional habits have the potential to result in poor Health-Related Quality of Life (HRQoL) in children and adolescents and vice versa [4]. Overall, a suboptimal diet being responsible for more deaths than any other risk factor globally is well-established knowledge [5]. Beyond laying the foundations of healthy bodily development, nutrition is suspected to influence children’s mental and social health. In preschool children, covering nutritional needs has a vital role in brain development and, consequently, in psychological responses and social behaviors [6,7]. However, the same connection is only suggestive and becomes rather complex in school-aged children and adolescents, particularly concerning mental health, where the limited evidence is accompanied by mixed associations between healthy dietary patterns and mental health [8]. Finally, another vital aspect of a student’s life, school-related wellbeing, is influenced by various dietary behaviors, with evident consequences of poor nutrition in school performance [9,10,11,12,13].

The Mediterranean diet is a universally recognized healthy dietary pattern with well-established health benefits in limiting the risk of non-communicable chronic diseases [14]. There is a growing body of evidence that adherence to the Mediterranean diet can benefit the adult population’s quality of life, particularly in relation to mental health [15]. In particular, two recent systematic reviews have provided evidence of a plausible connection between diet quality in general and Mediterranean diet adherence [4,16] with quality of life in children and adolescents. However, when examining the included studies, inconsistent findings regarding the different subscales of QoL are evident. Moreover, no evidence indicates that increasing Mediterranean diet adherence can positively affect the total HRQoL and consequently provide a dose–response relationship, a relationship heavily advocated in the aforementioned review [4].

In light of the gaps found in the existing literature, this study aims to evaluate the association of Mediterranean diet adherence with HRQoL in a large sample of school-aged children and adolescents by utilizing the database of the “DIATROFI” Program implemented in Greece. The DIATROFI program is a school-based food assistance initiative to reduce the limited access to adequate healthy food resulting from economic hardships in Greece. This is achieved by providing students with nutritious traditional Mediterranean diet-based daily meals and promoting healthy eating habits [17]. Two a priori research hypotheses have been examined here: a. the level of adherence to the Mediterranean diet is strongly associated with children and adolescents’ overall HRQoL as well as its specific subscales; b. improving the dietary habits of students coming from families living in underprivileged regions via a school-based food assistance program results in significant improvements in their HRQoL.

## 2. Materials and Methods

### 2.1. Study Design

The DIATROFI Program launched in 2012 and, without missing a single school year up till now, has offered school-based meals to more than 110,000 children. This food assistance initiative provides nutritious daily meals following the principles of the Mediterranean diet to students in pre-primary (kindergarten), primary, and secondary schools located in socially disadvantaged regions of Greece. The core aims of this initiative are to address students’ ever-increasing limited access to adequate healthy food while promoting healthy eating habits and behaviors.

The school-based sampling procedure can be found in detail in another publication [18] but, in a nutshell, researchers accounted for the school area’s socioeconomic deprivation level and access to adequate healthy food following a mixed methods approach. Overall, the included schools incorporate students mostly from low socioeconomic areas.

In the present work, we used data retrieved during the 2021–2022 school year with a one school year observation period.

### 2.2. Study Sample

In total, *n* = 152 schools and *n* = 5750 students across 4 main Greek regions (i.e., Attica, Macedonia, Thrace, and Central Greece) participated in the program in the 2021–2022 school year. The response rate at the baseline measurement (2021) was *n* = 3774 students (57.8% questionnaire response rate). There were *n* = 621 students excluded from the current study due to missing data related to their dietary habits, for a final sample size of *n* = 3153 students that was used to cross-sectionally associate students’ level of adherence to the Mediterranean diet with their HRQoL.

The 1-year follow-up was performed in May–June 2022 with *n* = 2455 students participating (42.7% questionnaire response rate). There were *n* = 1299 students excluded from the analysis due to missing data related to their dietary habits, for a final sample size of *n* = 1146 students that was used to prospectively associate students’ dietary trajectory (i.e., modification of dietary habits) with 1-year changes in their HRQoL.

### 2.3. Bioethics

The DIATROFI Program received ethical approval from the National and Kapodistrian University of Athens’ Bioethics Committee and the Ethical Committee of Prolepsis Institute (13416-10/2021). The program is being conducted in compliance with the Declaration of Helsinki and adheres to all relevant institutional regulations concerning the ethical use of human subjects and volunteers. Prior to participating in the program, all parents and school personnel were presented with a comprehensive information letter outlining the program’s objectives, implementation, and methodology. All questionnaires were completed anonymously, with informed consent obtained from parents prior to their participation.

### 2.4. Baseline and Follow-Up Assessment

#### 2.4.1. Data Collection

At the beginning (September–October 2021) and the end of the school year (May–June 2022), all participating schools received an anonymized questionnaire for each student. A parent (or another guardian if a parent was unable to answer) completed the questionnaire. As the questionnaires were anonymized, a set of sociodemographic characteristics were used to correctly identify and match the baseline and follow-up responses corresponding to each individual student.

#### 2.4.2. Students and Parental Sociodemographic Characteristics

Parents were asked at baseline (and follow-up) to report their own and their partner’s (biological parent) age, sex, country of birth, highest educational attainment, employment status, and the socioeconomic affluency of the family. The latter was assessed with the Family Affluence Scale (FAS) [19], comprising four questions. A three-point ordinal scale was used, where scores of 0–2, 3–5, and 6–9 were used to represent low-, moderate-, and high-socioeconomic affluence, respectively. Parental educational status was categorized as low (education: <9 years), moderate (10–12 years), and high (>12 years). Parents also reported their child’s age, sex, weight, height, and physical activity level (hours of extracurricular exercise/week). Body Mass Index (BMI) classification in students followed the standard definition [20].

#### 2.4.3. HRQoL Assessment

To evaluate students’ HRQoL, researchers utilized the PedsQL questionnaire, validated for the Greek population [21]. The questionnaire consists of 23 items categorized into four Generic Core Scales: physical, emotional, social, and school functions. Each item presents a scenario that a child may struggle with and has five response options, ranging from “never an issue” to “almost always an issue”. The Generic Core Scales, as well as the total HRQoL, are scored on a scale of 0 to 100, with higher scores indicating better HRQoL. As there are no established national thresholds, having poor HRQoL (for the total score and each subscale) was established as having a score less than the sample’s median. Though this categorization does not represent the actual number of students with poor health, it is valuable in examining the association of HRQoL with different characteristics. The lack of improvement between baseline and follow-up scores was classified as a decrease or no improvement in HRQoL score. The reliability and fit of the factorial structure of the PedsQL in the DIATROFI database were evaluated in a previous publication [18], which confirmed the tool’s reliability.

#### 2.4.4. Mediterranean Diet Adherence

The students’ dietary behavior was assessed at baseline and follow-up using the KIDMED questionnaire [22], which evaluates their adherence to the highly advocated, in Greece, Mediterranean dietary pattern. It comprises 16 questions examining the consumption of various food items/groups and breakfast consumption and is scored on a range of −4 to 12 (12 indicates optimal Mediterranean diet adherence). The level of adherence was classified into low (score: ≤3), moderate (4–7), and high (≥8) [22]. When comparing the baseline and follow-up adherence to the Mediterranean diet, four categories surfaced: (a) “Poor–stable” (indicating that the adherence was classified as poor at baseline and did not improve at follow-up); (b) moderate/high–stable (“MH–stable”), indicating that initial adherence was either moderate or high and did not improve at follow-up; (c) “Improved adherence”, indicating that the adherence was classified either as poor at baseline and moderate or high at follow-up or moderate at baseline and high at follow-up; and (d) “Worsen adherence”, indicating that the adherence was classified either as high at baseline and moderate or poor at follow-up or moderate at baseline and poor at follow-up.

### 2.5. Statistical Analysis

Categorical variables and continuous variables are presented as relative frequencies (%) and mean values (standard deviation), respectively. Normality was evaluated graphically (i.e., histograms, p-p plots, boxplots, q-q plots). The Student’s *t*-test was used to examine differences in KIDMED and HRQoL scores between two groups and one-way analysis of variance was used to examine differences in KIDMED and HRQoL scores between at least three different groups and the Bonferroni correction in the case of post hoc analysis. Similarly, chi-square analysis was performed to compare differences across categorical variables. A paired *t*-test was utilized to compare KIDMED and HRQoL scores between baseline and follow-up.

The median quality-of-life scores (HRQoL, physical function, emotional function, social function, school function) were used to classify students into two groups (below and above median). Multi-adjusted logistic regression models were utilized to explore the association between the level of adherence to the Mediterranean diet and the likelihood of having an HRQoL score below the median value at baseline. In an effort to reduce the bias in the resulting associations, all models were adjusted for a variety of confounders with evident existing connection with HRQoL (i.e., age, sex, family SES, BMI classification, physical activity).

Similar models were addressed to compare the association between changes in KIDMED score with the likelihood of improving HRQoL scores. Students were grouped in regard to their KIDMED classification at baseline and follow-up as worsen (from high to poor/moderate or moderate to poor), poor–stable (poor adherence in baseline and follow-up), MH–stable (MH adherence in baseline and follow-up), or improved (from poor to MH or moderate to high). A change in KIDMED score was defined as score at follow-up − score at baseline (higher values in score change indicate higher improvement). For each quality-of-life subscale score (HRQoL, physical function, emotional function, social function, school function), a score difference was calculated (score at follow-up − score at baseline) and then recoded into a two-group categorical variable indicating improvement (values > 0) or non-improvement (values ≤ 0) in quality of life.

All logistic regression models present their results as odds ratios (OR) and their corresponding 95% confidence intervals (95%CI). The Statistical Package for Social Sciences (IBM SPSS, Chicago) version 20.0 and the *p*-value threshold of significance (≤0.05) were used.

## 3. Results

### 3.1. Baseline Mediterranean Diet Adherence and HRQoL

In total, poor adherence to the Mediterranean diet accounted for almost one-fifth (19.7%) of the study sample, while moderate and high adherence accounted for more than a half (55.2%) and a fourth (25.1%) of the study sample, respectively. Sociodemographic characteristics for each group are presented in Supplementary Table S1. Students’ level of adherence to the Mediterranean diet and HRQoL according to their sociodemographic characteristics are presented in Table 1. Students of female sex, normal weight, or higher physical activity level reported a higher level of adherence to the Mediterranean diet (*p* < 0.05). Significantly elevated KIDMED and HRQoL scores were also recorded for students with better family socioeconomic affluency, parental income status, and educational level. Students in the group of poor HRQoL had a lower average KIDMED score compared with the group of good HRQoL. Similar results were observed for physical, emotional, social, and school functions (all *p*-values < 0.001).

The parent-perceived quality-of-life measurements of children and adolescents who participated in the present study are presented in Table 2. Overall, students with low adherence to the Mediterranean diet presented the worst HRQoL score (84.8 [13.8]), followed by students with moderate (89.0 [11.3]) and high adherence (90.2 [13.0]) to this pattern (*p* for trend < 0.001). A similar trend was observed in regard to physical, emotional, social, and school functions (all *p*-values < 0.001). The total HRQoL score, along with all four-dimensional scores (physical, emotional, social, and school functions), was lower than the median score in the majority of students with poor Mediterranean diet adherence, while the opposite was evident in subgroups with moderate and high levels of adherence (all *p*-values < 0.001).

In Table 3 multi-adjusted logistic regression analysis was performed to assess the association between students’ level of adherence to the Mediterranean diet and their likelihood of having an HRQoL score below the median value. Significant positive associations were revealed in all crude models, and these were retained across all models, even after adjusting for age, sex, family socioeconomic affluency, BMI classification, and physical activity. In particular, in the fully adjusted models, students with moderate or high adherence to the Mediterranean diet were 44% less likely to be assigned to a group of HRQoL below the median compared with students in low Mediterranean diet adherence (OR = 0.56, 95%CI = 0.44, 0.70). Further analysis on separate parameters of HRQoL revealed a similar effect of adherence to the Mediterranean diet on students’ physical, emotional, social, and school functions (all *p*-values < 0.05).

### 3.2. Mediterranean Diet Adherence and HRQoL from Baseline to Follow-Up

After a 1-year follow-up, different patterns of changes in students’ dietary habits were observed. In particular, 21.4% of students that started with poor or moderate level of adherence to the Mediterranean diet transitioned to a higher KIDMED score after 1 year. The majority, 64.6%, reported the same level (12.1% with poor adherence and 52.5% with moderate or high). Only 14% of students reported worse dietary habits in the follow-up period. The sociodemographic characteristics for each group are presented in Supplementary Table S2. An increase in both students’ KIDMED and HRQoL scores was evident during follow-up, as presented in Table 4 (all *p*-values < 0.001). Students with improved HRQoL, as well as emotional and social functions scores, reported a higher increment in the KIDMED score during follow-up (i.e., compared with students who did not improve their HRQoL at follow-up, students with improved HRQoL had increased their KIDMED score 1.5 times more) (all *p*-values < 0.05).

Multi-adjusted logistic regression models evaluating the association between changes in the students’ level of adherence to the Mediterranean diet and the likelihood of improving their quality of life at follow-up are provided in Table 5. Improvement in KIDMED score appeared to correlate with higher odds of improving their HRQoL, as well as emotional and social functions, in the crude models. After additional adjustments for age, sex, family socioeconomic affluency, physical activity, and BMI classification, the association remained. In more detail, a one-point increase in KIDMED score was associated with 9% higher likelihood of reporting an improvement of HRQoL (OR_HRQoL_ = 1.09, 95%CI = 1.02, 1.17), 9% higher likelihood of emotional functioning (OR_emotional_ = 1.09, 95%CI = 1.02, 1.17), and 13% higher likelihood of social functioning (OR_social_ = 1.13, 95%CI = 1.05, 1.22). In the case of physical and school functions, no significant associations were revealed.

Focusing on dietary trajectories, MH–stable or students who increased their level of adherence to the Mediterranean diet at follow-up were more likely to improve their HRQoL (OR_MH–stable_ = 1.72, 95%CI = 1.05, 2.80; OR_Improved_ = 1.57, 95%CI = 1.02, 2.44) and emotional function (OR_MH–stable_ = 1.73, 95%CI = 1.03, 2.92; OR_Improved_ = 1.77, 95%CI = 1.10, 2.83) compared with the low–stable group, after adjusting for age, sex, family socioeconomic affluency, and physical activity. BMI seemed to have a moderating effect either attenuating the examined association (in the case of emotional function) or resulting in lost significance (i.e., in the case of HRQoL).

## 4. Discussion

The present article demonstrates that the level of adherence to a healthy dietary pattern, the Mediterranean diet, is closely linked with all dimensions of HRQoL in a sample of Greek children. Overall, the majority of students demonstrated high HRQoL accompanied by moderate adherence to the Mediterranean diet pattern. Students with moderate or high adherence to the Mediterranean diet reported higher physical, emotional, social, and school functions compared to those with low adherence. Having moderate or high adherence to the Mediterranean diet was associated with a greater chance of having higher-than-average HRQoL, physical, emotional, social, and school functions, even after accounting for confounders. The program was successful at improving the student’s dietary quality and HRQoL. A one-point increase in KIDMED score (from baseline till follow-up) was associated with a greater chance of having improved HRQoL, emotional, and social functions at follow-up. This study confirms the connection of Mediterranean diet adherence and HRQoL in children and adolescents. However, it is one of the few studies that has examined the connection between dietary trajectories (including improvement or worsening) and HRQoL change, underscoring the perplexing effect of dietary improvement following the Mediterranean diet principles on mental and, potentially, on social wellbeing.

In our study, a large representation of the Greek youth population indicated that approximately one in five children have poor and half have moderate adherence to the Mediterranean dietary pattern. The majority of studies conducted in the Greek population has indicated a similar prevalence of children with moderate adherence, but the prevalence of poor adherence vastly differs across studies [23,24,25]. Recent studies conducted on the Greek population have indicated a higher prevalence of low adherence in adolescents [25], and even lower in children and preadolescents [24]. Assessing the HRQoL of our sample may be difficult as no cut-off criteria have been set for the PedsQL questionnaire in regard to the general healthy child population in Greece. Comparing our results with the Greek population is almost impossible as recent studies have used mostly different tools to assess HRQoL [16]. Studies have indicated a vast range of scores, attributed to cultural, age, and socioeconomic differences [16,26,27,28,29].

Our research has yielded evidence of a correlation between following the Mediterranean diet and all aspects of children’s HRQoL. Additionally, we have identified a link between the degree of improvement in adherence to the Mediterranean diet and short-term enhancement of HRQoL, in a dose–response manner. The association between Mediterranean diet and HRQoL in young people has been the main focus of two recent systematic reviews, which have found that most studies generally support this relationship [4,16]. Our results are in line with two recent studies, with one indicating a dose–response association between Mediterranean diet adherence and HRQoL in children [25,30] and another indicating an association with all HRQoL dimensions in adolescents [31]. Similar findings were also apparent during social isolation in Spanish and Brazilian children and adolescents [32]. However, when examining the association with each dimension of HRQoL, many disparities are evident across studies [4,16]. Moreover, these results were based on studies including mostly preadolescents and adolescents [4,16], while the child population was limited and did not assess the connection between Mediterranean diet and all subscales of HRQoL [25,30]. Our study stands among the very few that have examined the connection of HRQoL and its dimensions with Mediterranean diet adherence in a predominantly young population. On the top of this, we progressively examined the change in dietary habits within a one school year observation period in relation to the HRQoL change, taking into account various potential confounders that might intervene to the observed associations.

In regard to emotional wellbeing, a more apparent dose–response connection was evident. The Mediterranean diet in children has been linked with lower chances of depressive symptomatology and improved cognitive function [33,34]. Nutritional adequacy characterizes this dietary pattern, which is a requirement for healthy cognitive development during childhood [35,36]. Moreover, the DIATROFI food assistance program has proven to alleviate food insecurity and hunger in children, with both well-known mental stressors and predictors of deficiencies [37]. Finally, although the connection between the Mediterranean diet and excess weight or adiposity is a subject of debate [38,39], methodologically sound interventions based on the Mediterranean diet may reduce obesity rates across obese children [40]. By doing so, a significant mental stressor, the obesity-related stigma, may decrease, contributing to higher mental health [41].

On the other hand, our results highlighted the connection between social wellbeing and diet in children. Though a dose–response connection was evident with Mediterranean diet adherence, when a child improved their adherence classification, no improvement in social function was apparent. Studies have indicated that malnutrition at the preschool age is associated with impaired social behavior, potentially due to the mediating effect of poor neurocognitive function [6]. Additionally, obesity-related stigma may pose a barrier in forming friendships and lead to the social isolation of children [41]. A study conducted among adolescents found that the correlation between Mediterranean diet adherence and perceived happiness is mediated by perceived social acceptance, translating to feeling respected and accepted by peers while avoiding not feeling bullied [31]. Considering that the youth sample of the present work came from underprivileged regions, other socioeconomically determined factors may have attenuated the beneficial effect of nutrition on quality of life.

Physical and school functions were not significantly improved after improving Mediterranean diet adherence, even if a cross-sectional association was observed. Although the benefits of dietary improvement on disease prevention and health improvement are evident in children, we believe that the immediate health benefit in a sample of healthy children may not be noticeable within one school year. As for school function, optimal dietary habits can contribute to optimal cognitive development, working memory, and school performance in children and adolescents [34,42,43]. Following such a dietary pattern may be linked with improved sleeping habits, which mediates the connection between Mediterranean diet and school performance in children [43]. Evidence of a significant connection between baseline measures of academic performance in youth and Mediterranean diet adherence [43] has partially confirmed our findings. However, school wellbeing and engagement can be influenced by factors not accounted for in our study, which may lead to fluctuations in school-related wellbeing and performance throughout a school year. For instance, students with a fear of failure, often attributed to parental expectations, and low self-efficacy may indicate lower school engagement [44], especially during the highly stressful final examination period that was our follow-up period. Prospective studies should examine this connection in more depth, comparing school performance and functioning at the same time point each school year.

### Strengths and Limitations

This study’s unique focus on the connection between changes in Mediterranean diet adherence and changes in HRQoL in children and adolescents sets it apart from the vast majority of HRQoL research, going beyond their cross-sectional association to an analysis that presents the pattern of HRQoL modifications in early life stages according to observed 1-year dietary trajectories. Thus, the current analysis provides novel insights into their complex connection in youth. Nevertheless, as in every study, this research has some limitations. Firstly, we were unable to include data from all the subjects participating in this study at baseline and an even smaller sample was compiled with both baseline and follow-up data available. Secondly, the cross-sectional design of our initial analysis and the analysis of changes in HRQoL and Mediterranean diet adherence prevents us from establishing causal relations, even if there is some evidence provided of a dose–response connection. Moreover, this dose–response connection accounts for only one school year, which might prove insufficient in ensuring all outcomes had sufficient time to be noticeable and provide evidence of long-term results. Thirdly, the PedsQL does not include other aspects of children’s wellbeing, such as material and cultural wellbeing, while educational wellbeing and cognitive development are partially assessed by its school function dimension [1]. Fourthly, the division of poorer or higher-than-average HRQoL is not indicative of the number of children with low HRQoL but occurs as a limitation of the absence of cut-off values for the PedsQL and the reasonably high HRQoL scores in our sample of healthy youth. Lastly, students, particularly those in secondary schools, may experience elevated stress and poor social health at the end of the school year due to the stress related to final examinations [45]. Yet, our study has indicated that regardless of the student’s age, an increase in Mediterranean diet adherence is linked with an increase in both emotional and social health.

## 5. Conclusions

Our evidence suggests a substantial connection between Mediterranean diet and HRQoL in children. Improvement in Mediterranean diet adherence may be beneficial towards ameliorating HRQoL in children, particularly their mental and social health wellbeing. Longitudinal studies could strengthen these findings and uncover the precise impact on different aspects of their overall wellbeing. Encouraging healthy dietary habits in children and adolescents is crucial since the benefits are not restricted to physical health but also extend to their general wellbeing. Implementing school-based initiatives to address the declining dietary quality in young individuals may play a critical role in promoting their overall wellbeing.

## Figures and Tables

**Table 1:** Students’ level of adherence to the Mediterranean diet and parent-perceived quality-of-life measurements in students who participated in the DIATROFI Program according to students’ sociodemographic characteristics at baseline.

	Total Sample, %	KIDMED, Score (Range [−4, 12])	*p*-Value	HRQoL, Score (Range [0, 100])	*p*-Value
Total sample	n = 3153				
Students’ characteristics		5.61 (2.61)	-	88.5 (12.4)	-
Sex					
Boys	51.2	5.53 (2.66)	0.020	88.0 (12.5)	<0.001
Girls	48.8	5.76 (2.51)		89.2 (12.0)	
Students’ educational attainment					
Pre-primary school (kindergarten)	23.4	5.65 (2.75)	0.206	87.7 (11.5)	<0.001
Primary school	74.4	5.62 (2.57)		88.9 (12.4)	
Secondary school	2.2	5.07 (2.56)		82.7 (17.4)	
BMI classification					
Underweight	12.0	5.64 (2.52)	0.023	88.2 (12.7)	0.063
Normal	52.8	5.73 (2.59)		89.3 (11.8)	
Overweight/Obese	35.2	5.45 (2.61)		88.1 (12.5)	
Physical activity (weekly)					
<2 h	53.5	5.27 (2.77)	<0.001	87.2 (13.3)	<0.001
>2 h	46.5	6.00 (2.36)		90.2 (10.9)	
Family characteristics					
Parental income status					
Both parents with income	51.3	5.78 (2.37)	<0.001	89.8 (11.4)	<0.001
One parent with income	42.4	5.77 (2.46)		88.7 (12.1)	
Both parents without income	6.3	3.74 (3.67)		83.2 (13.7)	
Paternal educational level					
Low	30.1	5.45 (2.82)	0.004	86.6 (13.8)	<0.001
Moderate	40.6	5.70 (2.43)		89.7 (11.3)	
High	29.3	5.85 (2.36)		90.1 (10.6)	
Maternal educational level					
Low	21.8	4.98 (3.16)	<0.001	85.1 (14.0)	<0.001
Moderate	30.2	5.77 (2.45)		89.7 (11.3)	
High	48.0	5.78 (2.38)		89.6 (11.4)	
Family socioeconomic status					
Low	23.7	4.68 (3.03)	<0.001	85.6 (13.7)	<0.001
Moderate	56.3	5.81 (2.39)		89.4 (11.6)	
High	20.0	6.15 (2.38)		89.9 (12.0)	
Students’ quality-of-life measurements					
Above median HRQoL score	47.0	6.05 (2.45)	<0.001	-	-
Below median HRQoL score	53.0	5.17 (2.67)		-	
Above median physical function score	54.0	5.97 (2.52)	<0.001	-	-
Below median physical function score	46.0	5.14 (2.63)		-	
Above median emotional function score	52.8	5.87 (2.54)	<0.001	-	-
Below median emotional function score	47.2	5.29 (2.63)		-	
Above median social function score	52.7	5.97 (2.45)	<0.001	-	-
Below median social function score	47.3	5.17 (2.70)		-	
Above median school function score	50.6	6.02 (2.44)	<0.001	-	-
Below median school function score	49.4	5.15 (2.69)		-	

Parental educational level was defined as low (≤9 years of education), moderate (10–12 years of education), and high (>12 years of education). Family socioeconomic status was defined according to the Family Affluence Scale, i.e., low (FAS = 0–2), middle (FAS = 3–5), and high (FAS = 6–9). Adherence to the Mediterranean diet was defined according to KIDMED score. Data are presented as mean (standard deviation) for KIDMED and HRQoL scores and % of the corresponding sample for categorical variables. The median quality-of-life scores (HRQoL, physical function, emotional function, social function, school function) were used to classify students into two groups (below and above median). The revised international IOTF BMI cut-offs according to the pooled LMS curves were used for BMI classification (underweight, normal, overweight, obese). *p*-values were obtained using Student’s *t*-test in the case of comparisons between two independent samples and categorical one-way analysis of variance in the case of comparisons among 3 or more independent samples. Abbreviations: International Obesity Task Force (IOTF); Lambda Mu and Sigma (LMS); Standard Deviation (SD); Body Mass Index (BMI); Health-Related Quality of Life score (HRQoL).

**Table 2:** Parent-perceived quality-of-life measurements in students according to their level of adherence to the Mediterranean diet at baseline.

	Total Sample	Students’ Level of Adherence to the Mediterranean Diet			
Students’ Quality-of-Life Measurements		Poor	Moderate	High	*p*-Value
*n*	3153	621	1742	790	
HRQoL, score (range [0, 100]), Mean (SD)	88.3 (12.6)	84.8 (13.8)	89 (11.3)	90.2 (13)	<0.001
Below median HRQoL score, %	53.0	62.9	52.8	42.3	<0.001
Physical function, score (range [0, 100]), Mean (SD)	90.7 (14.2)	88.4 (15)	91.3 (13)	91.9 (15.1)	<0.001
Below median physical function score, %	46.0	54.9	47.1	35.5	<0.001
Emotional function, score (range [0, 100]), Mean (SD)	84.9 (16.1)	82 (17.5)	85.1 (15.4)	87.2 (16.3)	<0.001
Below median emotional function score, %	47.2	54.4	47.2	39.5	<0.001
Social function, score (range [0, 100]), Mean (SD)	89.2 (16.6)	84.8 (18.9)	90.5 (15)	91.1 (15.8)	<0.001
Below median social function score, %	47.3	57.7	46.3	39.4	<0.001
School function, score (range [0, 100]), Mean (SD)	86.8 (16.4)	81.9 (18.5)	87.8 (14.8)	89.6 (16)	<0.001
Below median school function score, %	49.4	60.9	49.2	39.0	<0.001

Level of adherence to the Mediterranean diet was defined according to KIDMED score as follows: poor (≤3), moderate (4–7) and high (≥8). Students’ quality of life was measured via the Pediatric Quality of Life Inventory questionnaire (PedsQL) answered by students’ parents. The median quality-of-life scores (HRQoL, physical function, emotional function, social function, school function) were used to classify students into two groups (below and above median). Data are presented as mean (standard deviation) for normally distributed continuous variables (quality-of-life scores) and % of the corresponding sample for categorical variables. For the normally distributed variables (quality-of-life scores), *p*-values were obtained using one-way analysis of variance and the Bonferroni correction in the case of post hoc analysis. For the categorical variables, a chi-squared test was performed. Abbreviation: Health-Related Quality of Life (HRQoL).

**Table 3:** Logistic regression models evaluating the association between level of adherence to the Mediterranean diet and parent-perceived quality of life of students at baseline (*n* = 3153).

	Dependent Variable	Below Median HRQoL Score	Below Median Physical Function Score	Below Median Emotional Function Score	Below Median Social Function Score	Below Median School Function Score	
**Model 1**	**Students’ level of adherence to the Mediterranean diet**	**OR (95%CI)**	**OR (95%CI)**	**OR (95%CI)**	**OR (95%CI)**	**OR (95%CI)**	**Model** **adjusted for**
	Poor	ref	ref	ref	ref	ref	(crude model)
	Moderate/High	0.58 (0.48, 0.70) ***	0.63 (0.53, 0.76) ***	0.68 (0.57, 0.82) ***	0.58 (0.49, 0.69) ***	0.55 (0.46, 0.66) ***	
**Model 2**	**Students’ level of adherence to the Mediterranean diet**	**OR (95%CI)**	**OR (95%CI)**	**OR (95%CI)**	**OR (95%CI)**	**OR (95%CI)**	
	Poor	ref	ref	ref	ref	ref	Age, sex
	Moderate/High	0.58 (0.48, 0.71) ***	0.64 (0.53, 0.78) ***	0.72 (0.60, 0.87) ***	0.60 (0.49, 0.72) ***	0.56 (0.46, 0.68) ***	
**Model 3**	**Students’ level of adherence to the Mediterranean diet**	**OR (95%CI)**	**OR (95%CI)**	**OR (95%CI)**	**OR (95%CI)**	**OR (95%CI)**	
	Poor	ref	ref	ref	ref	ref	Model 2 + family SES, BMI classification, physical activity (hours/week)
	Moderate/High	0.56 (0.44, 0.70) ***	0.66 (0.53, 0.83) ***	0.73 (0.58, 0.91) **	0.61 (0.49, 0.77) ***	0.56 (0.45, 0.71) ***	

The revised international IOTF BMI cut-offs according to the pooled LMS curves were used for BMI classification (underweight, normal, overweight, obese). Level of adherence to the Mediterranean diet was defined according to KIDMED score as follows: poor (≤3), moderate (4–7), and high (≥8). Students’ quality of life was measured via the Pediatric Quality of Life Inventory questionnaire (PedsQL) answered by students’ parents. The median quality-of-life scores (HRQoL, physical function, emotional function, social function, school function) were used to classify students into two groups (below and above median). *** *p* < 0.01; ** *p* < 0.05. Abbreviations: Body Mass Index (BMI); Health-Related Quality of Life (HRQoL); Odds Ratio (OR); Socioeconomic level (SES); 95% Confidence Interval (95%CI); International Obesity Task Force (IOTF); Lambda Mu and Sigma (LMS); Moderate/High (MH).

**Table 4:** Changes in students’ level of adherence to the Mediterranean diet and parent-perceived quality of life from baseline to follow-up evaluation according to sociodemographic characteristics of students and their family.

	Baseline KIDMED, Score (Range [−4, 12])	Difference (Follow-Up − Baseline)	*p*-Value	Baseline HRQoL, Score (Range [0, 100])	Difference (Follow-Up − Baseline)	*p*-Value
Total sample (n = 1146)	5.55 (2.53)	+0.32 (2.14)	<0.001	89.7 (12.0)	+1.3 (10.6)	<0.001
Students’ characteristics						
Sex						
Boys	5.48 (2.58)	0.25 (2.19) *	0.223	89.3 (12.3)	1.4 (11.3) *	0.891
Girls	5.61 (2.53)	0.41 (2.10) *		90.1 (12.1)	1.3 (10.5) *	
Students’ educational attainment						
Pre-primary school (kindergarten)	5.20 (3.05)	0.28 (2.11) *		87.9 (11.6)	0.7 (8.2)	
Primary school	5.69 (2.34)	0.32 (2.12) *	0.444	90.6 (11.6)	1.7 (11.2) *	0.219
Secondary school	4.78 (2.54)	0.72 (2.68)		80.5 (19.9)	−3.1 (11.0)	
BMI classification						
Underweight	5.59 (2.51)	0.68 (2.18) *	0.761	90.8 (11)	0.6 (10.2)	0.996
Normal	5.65 (2.64)	0.32 (2.08) *		90.3 (11.9)	1.0 (11.2)	
Overweight/Obese	5.44 (2.50)	0.22 (2.14)		88.4 (13.4)	2.1 (11.2) *	
Physical activity (weekly)						
<2 h	5.11 (2.73)	0.35 (2.06) *	0.928	88.8 (12.8)	0.6 (10.5)	0.026
>2 h	5.95 (2.26)	0.33 (2.20) *		90.2 (11.5)	2.0 (11.0) *	
Family characteristics						
Parental income status						
Both parents with income	5.73 (2.30)	0.30 (2.15) *		90.3 (11.8)	2.1 (10.7) *	
One parent with income	5.72 (2.42)	0.32 (2.02) *	0.318	90.4 (10.8)	0.9 (9.8)	0.518
Both parents without income	3.47 (3.77)	0.37 (2.66)		82.1 (14.8)	−2.2 (9.9)	
Paternal educational level						
Low	5.02 (2.98)	0.16 (2.21)		87.5 (13.9)	1.1 (12.4)	
Moderate	5.79 (2.31)	0.35 (2.09) *	0.413	90.5 (11.7)	1.8 (11.1) *	0.418
High	5.78 (2.30)	0.43 (2.15) *		90.9 (10)	1.0 (7.2) *	
Maternal educational level						
Low	4.47 (3.14)	0.23 (2.35)		86 (13.6)	−0.3 (9.8)	
Moderate	5.81 (2.46)	0.35 (2.00) *	0.016	90.3 (12)	1.4 (10.8) *	0.048
High	5.75 (2.27)	0.35 (2.10) *		90.5 (11.1)	1.8 (10.2) *	
Family socioeconomic status						
Low	4.43 (3.09)	0.36 (2.17) *		87.6 (11.9)	−0.1 (10.1)	
Moderate	5.77 (2.32)	0.30 (2.17) *	0.303	90 (11.8)	1.3 (10.2) *	0.218
High	5.92 (2.32)	0.29 (1.94) *		90.2 (13.1)	3.0 (12.0) *	
Changes in students’ quality of life						
Improved HRQoL	5.56 (2.37)	0.50 (2.20) *	0.016	-	-	-
Not improved HRQoL	5.48 (2.66)	0.33 (2.08) *		-	-	
Improved physical function	5.42 (2.29)	0.37 (2.20) *	0.770	-	-	-
Not improved physical function	5.57 (2.64)	0.33 (2.08) *		-	-	
Improved emotional function	5.6 (2.26)	0.51 (2.09) *	0.037	-	-	-
Not improved emotional function	5.47 (2.68)	0.24 (2.14) *		-	-	
Improved social function	5.28 (2.41)	0.58 (2.23) *	0.039	-	-	-
Not improved social function	5.59 (2.56)	0.27 (2.08) *		-	-	
Improved school function	5.52 (2.49)	0.38 (2.34) *	0.700	-	-	-
Not improved school function	5.52 (2.55)	0.32 (2.02) *		-	-	

Parental educational level was defined as low (≤9 years of education), moderate (10–12 years of education), and high (>12 years of education). Family socioeconomic status was defined according to the Family Affluence Scale, i.e., low (FAS = 0–2), middle (FAS = 3–5), and high (FAS = 6–9). The revised international IOTF BMI cut-offs according to the pooled LMS curves were used for BMI classification (underweight, normal, overweight, obese). Students’ quality of life was measured via the Pediatric Quality of Life Inventory questionnaire (PedsQL) answered by students’ parents. Level of adherence to the Mediterranean diet was defined according to KIDMED score. Changes in KIDMED and quality-of-life scores were defined as the difference in follow-up score − baseline score. For each quality-of-life score (HRQoL, physical function, emotional function, social function, school function), the generated score difference was then recoded into a two-group categorical variable indicating improvement (values > 0) or non-improvement (values ≤ 0) in quality of life. Data are presented as mean (standard deviation). *p*-Values were obtained using Student’s *t*-test for categorical variables with two groups, one-way analysis of variance for more than two groups, and the Bonferroni correction in the case of post hoc analysis and paired Student’s *t*-test for pre-post analysis. * *p* < 0.05 in regard to paired Student’s *t*-test within each group. Abbreviations: International Obesity Task Force (IOTF); Lambda Mu and Sigma (LMS); Standard Deviation (SD); Body Mass Index (BMI); Moderate/High (MH); Health-Related Quality of Life score (HRQoL).

**Table 5:** Logistic regression models evaluating the association between trajectories in the level of adherence to the Mediterranean diet and the odds of parent-perceived quality-of-life improvement of students during the follow-up period.

	Dependent Variable	Improvement in HRQoL	Improvement in Physical Function	Improvement in Emotional Function	Improvement in Social Function	Improvement in School Function	
**Model 1**	**Adherence to the Mediterranean diet trajectories**	**OR (95%CI)**	**OR (95%CI)**	**OR (95%CI)**	**OR (95%CI)**	**OR (95%CI)**	**Model adjusted for**
	Poor–stable	ref	ref	ref	ref	ref	(crude model)
	Worsen	1.26 (0.79, 2.02)	1.34 (0.81, 2.22)	1.20 (0.72, 1.98)	0.72 (0.41, 1.25)	1.12 (0.68, 1.83)	
	Improved	1.56 (1.07, 2.29) **	1.49 (0.99, 2.25) *	1.66 (1.11, 2.49) **	0.91 (0.59, 1.39)	0.95 (0.64, 1.43)	
	MH–stable	1.85 (1.21, 2.84) **	1.54 (0.97, 2.42) *	1.70 (1.08, 2.67) **	0.99 (0.61, 1.61)	0.99 (0.63, 1.55)	
	**Change in KIDMED score (units)**	1.07 (1.01, 1.13) **	1.01 (0.95, 1.07)	1.06 (1.01, 1.12) **	1.07 (1.01, 1.14) **	1.01 (0.95, 1.07)	
**Model 2**	**Adherence to the Mediterranean diet trajectories**	**OR (95%CI)**	**OR (95%CI)**	**OR (95%CI)**	**OR (95%CI)**	**OR (95%CI)**	
	Poor–stable	ref	ref	ref	ref	ref	Age, sex
	Worsen	1.19 (0.73, 1.96)	1.25 (0.74, 2.12)	1.29 (0.76, 2.19)	0.64 (0.36, 1.14)	1.27 (0.75, 2.13)	
	Improved	1.62 (1.09, 2.42) **	1.51 (0.99, 2.32) *	1.86 (1.21, 2.87) **	0.91 (0.59, 1.42)	1.04 (0.68, 1.59)	
	MH–stable	1.82 (1.16, 2.87) **	1.56 (0.97, 2.52) *	1.79 (1.10, 2.90) **	0.96 (0.58, 1.59)	1.11 (0.68, 1.79)	
	**Change in KIDMED score (units)**	1.08 (1.02, 1.15) ***	1.01 (0.95, 1.07)	1.06 (1.01, 1.13) **	1.08 (1.01, 1.16) **	1.02 (0.96, 1.08)	
**Model 3**	**Adherence to the Mediterranean diet trajectories**	**OR (95%CI)**	**OR (95%CI)**	**OR (95%CI)**	**OR (95%CI)**	**OR (95%CI)**	
	Poor–stable	ref	ref	ref	ref	ref	Model 2 + family SES, physical activity (h/week)
	Worsen	1.11 (0.65, 1.89)	1.11 (0.63, 1.96)	1.17 (0.66, 2.07)	0.58 (0.31, 1.09) *	1.09 (0.62, 1.90)	
	Improved	1.57 (1.02, 2.44) **	1.35 (0.85, 2.14)	1.77 (1.10, 2.83) **	0.88 (0.55, 1.43)	0.93 (0.58, 1.48)	
	MH–stable	1.72 (1.05, 2.80) **	1.40 (0.84, 2.34)	1.73 (1.03, 2.92) **	0.99 (0.58, 1.69)	0.94 (0.56, 1.58)	
	**Change in KIDMED score (units)**	1.08 (1.02, 1.15) **	1.01 (0.95, 1.08)	1.07 (1.01, 1.14) **	1.11 (1.03, 1.19) ***	1.02 (0.95, 1.09)	
**Model 4**	**Adherence to the Mediterranean diet trajectories**	**OR (95%CI)**	**OR (95%CI)**	**OR (95%CI)**	**OR (95%CI)**	**OR (95%CI)**	
	Poor–stable	ref	ref	ref	ref	ref	Model 3 + BMI classification
	Worsen	1.01 (0.57, 1.78)	1.05 (0.58, 1.92)	1.01 (0.55, 1.88)	0.55 (0.28, 1.07)	1.01 (0.55, 1.82)	
	Improved	1.4 (0.88, 2.23)	1.27 (0.78, 2.07)	1.73 (1.05, 2.86) **	0.84 (0.50, 1.39)	0.86 (0.52, 1.40)	
	MH–stable	1.59 (0.95, 2.67) *	1.27 (0.73, 2.19)	1.70 (0.98, 2.96) *	0.98 (0.56, 1.73)	0.89 (0.52, 1.55)	
	**Change in KIDMED score (units)**	1.09 (1.02, 1.17) **	1.02 (0.95, 1.09)	1.09 (1.02, 1.17) **	1.13 (1.05, 1.22) ***	1.02 (0.95, 1.10)	

The revised international IOTF BMI cut-offs according to the pooled LMS curves were used for BMI classification (underweight, normal, overweight, obese). Level of adherence to the Mediterranean diet was defined according to KIDMED score as follows: poor (≤3), moderate (4–7), and high (≥8). Students’ quality of life was measured via the Pediatric Quality of Life Inventory questionnaire (PedsQL) answered by students’ parents. Students were classified in regard to their KIDMED classification at baseline and follow-up as follows: worsen (from high to poor/moderate or moderate to poor), poor–stable (poor adherence in baseline and follow-up), MH–stable (MH adherence in baseline and follow-up), or improved (from poor to MH or moderate to high). Change in KIDMED score was defined as score at follow-up − score at baseline (higher values in score change indicate higher improvement). For each quality-of-life score (HRQoL, physical function, emotional function, social function, school function), a score difference was calculated (score at follow-up − score at baseline) and then recoded into a two-group categorical variable indicating improvement (values > 0) or non-improvement (values ≤ 0) in quality of life. *** *p* < 0.01; ** *p* < 0.05; * *p* < 0.10. Abbreviations: Body Mass Index (BMI); Health-Related Quality of Life (HRQoL); Odds Ratio (OR); Socioeconomic level (SES); 95% Confidence Interval (95%CI); International Obesity Task Force (IOTF); Lambda Mu and Sigma (LMS); Moderate/High (MH).

## Data Availability

The data presented in this study are available on request from the corresponding author.

## Supplementary Materials

The following supporting information can be downloaded at: https://www.mdpi.com/article/10.3390/nu15081829/s1. Table S1. Sociodemographic characteristics of students and their family in the total sample and according to students’ level of adherence to the Mediterranean diet. Table S2. Sociodemographic characteristics and trajectories in parent-perceived quality of life of matched students according to changes in students’ level of adherence to the Mediterranean diet.
