# Supplementary material for: Health-Related Quality of Life in Childhood and Adolescence: The Interrelation with Level of Adherence to Mediterranean Diet and Dietary Trajectories: Highlights from the DIATROFI Program (2021–2022)
Source: Nutrients. 2023 Apr 10;15(8):1829. doi: 10.3390/nu15081829 (PMC10142042; doi:10.3390/nu15081829)
Supplement: Supplementary file 1 [file nutrients-15-01829-s001.zip › nutrients-2322392-supplementary.pdf]

nutrients

MDPI

Table S1. Sociodemographic characteristics of students and their family in the total sample and according to students' level of adherence to the Mediterranean diet.

Characteristics of Students and Their Family	Total Sample	Students' Level of Adherence to Mediterranean Diet			
		**Poor**	**Moderate**	**High**	***p*-Value**
n	3153	621	1742	790	
**Students' characteristics**					
Age (years), Mean (SD)	7.8 (2.6)	8.1 (2.7)	7.7 (2.5)	7.6 (2.6)	<0.001
Boys, %	51.2	57.0	49.0	51.5	0.006
Students' educational attainment					
Pre-primary school (kindergarten)	23.4	21.9	22.7	26.1	0.060
Primary school	74.4	74.9	75.0	72.5	
Secondary school	2.2	3.2	2.2	1.4	
BMI classification, %					
Underweight	12.0	11.8	12.0	12.0	0.338
Normal	52.8	50.2	52.2	56.0	
Overweight/Obese	35.2	37.9	35.8	32.1	
Physical activity (weekly)					
<2 h	53.5	64.8	52.1	47.6	<0.001
>2 h	46.5	35.2	47.9	52.4	
**Family characteristics**					
Parental income status, %					
Both parents with income	51.3	46.0	53.3	50.8	<0.001
One parent with income	42.4	39.1	42.9	44.0	
Both parents without income	6.3	14.9	3.8	5.3	
Paternal educational level, %					
Low	30.1	35.8	27.7	31.2	<0.001
Moderate	40.6	41.0	40.7	40.1	
High	29.3	23.2	31.6	28.7	
Maternal educational level, %					
Low	21.8	31.7	18.3	21.7	<0.001
Moderate	30.2	27.5	31.2	30.0	
High	48.0	40.8	50.5	48.3	
Family socioeconomic status, %					
Low	23.7	38.4	20.5	19.1	<0.001
Moderate	56.3	46.5	59.8	56.1	
High	20.0	15.1	19.7	24.8	

Parental educational level was defined as low (≤9 years of education), moderate (10–12 years of education), and high (>12 years of education). Level of adherence to the Mediterranean diet was defined according to KIDMED score as follows: poor (≤3), moderate (4–7), and high (≥8). The revised international IOTF BMI cut-offs according to the pooled LMS curves were used for BMI classification (underweight, normal, overweight, obese). Data are presented as mean (standard deviation) for normally distributed continuous variables (age) and % of the corresponding sample for categorical variables. For the normally distributed variables (age), *p*-values were obtained using one-way analysis of variance and the Bonferroni correction in the case of post hoc analysis. For the categorical variables, a chi-squared test was performed. Abbreviations: International Obesity Task Force (IOTF); Lambda Mu and Sigma (LMS); Standard Deviation (SD); Body Mass Index (BMI).

Table S2. Sociodemographic characteristics and trajectories in parent-perceived quality of life of matched students according to changes in students' level of adherence to the Mediterranean diet.

	Total Sample	Students' Level of Adherence to Mediterranean Diet				
**Characteristics of Students and Their Family at Baseline**		**Worsen**	**Poor–Stable**	**MH–Stable**	**Improved**	***p*-Value**
n	1146	160	139	602	245	
Students' characteristics						
Age (years), Mean (SD)	7.7 (2.6)	8.1 (2.6)	7.8 (2.8)	7.5 (2.4)	8.1 (2.7)	0.009
Boys, %	50.0	54.5	58.6	49.1	44.0	0.042
Students' educational attainment						
Pre-primary school (kindergarten)	23.2	19.4	33.1	23.9	18.4	0.026
Primary school	74.0	77.5	63.3	73.9	78.0	
Secondary school	2.8	3.1	3.6	2.2	3.7	
BMI classification, %						
Underweight	11.4	6.2	9.7	11.3	16.1	0.176
Normal	54.5	55.3	58.7	54.5	51.3	
Overweight/Obese	34.2	38.5	31.6	34.2	32.6	
Physical activity (weekly)						
<2 h	50.2	46.5	72.9	47.6	46.2	<0.001
>2 h	49.8	53.5	27.1	52.4	53.8	
Family characteristics						
Parental income status, %						
Both parents with income	54.3	58.8	45.2	55.9	52.4	<0.001
One parent with income	40.1	35.8	32.5	41.4	44.0	
Both parents without income	5.6	5.4	22.2	2.8	3.6	
Paternal educational level, %						
Low	27.2	27.5	46.6	24.1	23.7	<0.001
Moderate	41.7	38.6	31.6	44.6	42.4	
High	31.1	34.0	21.8	31.3	33.9	
Maternal educational level, %						
Low	17.3	15.0	40.3	13.8	14.4	<0.001
Moderate	27.7	28.1	21.6	29.2	27.0	
High	55.1	56.9	38.1	57.1	58.7	
Family socioeconomic status, %						
Low	18.9	19.0	39.4	14.7	17.7	<0.001
Moderate	59.6	58.2	42.4	62.1	64.2	
High	21.5	22.9	18.2	23.2	18.1	
Trajectories in students' quality of life						
HRQoL (not improved), %	53.3	57.5	63.0	52.2	47.9	0.024
Physical function (not improved), %	65.3	66.5	72.7	64.0	63.4	0.238
Emotional function (not improved), %	62.8	68.0	71.7	60.5	59.9	*0.034*
Social function (not improved), %	76.4	80.4	74.6	76.4	74.8	0.586
School function (not improved), %	69.4	66.7	69.1	70.1	69.4	0.876

Parental educational level was defined as low (≤9 years of education), moderate (10–12 years of education), and high (>12 years of education). The revised international IOTF BMI cut-offs according to the pooled LMS curves were used for BMI classification (underweight, normal, overweight, obese). Students' quality of life was measured via the Pediatric Quality of Life Inventory questionnaire (PedsQL) answered by students' parents. The level of adherence to the Mediterranean diet was defined according to KIDMED score as follows: poor (≤3), moderate (4–7), and high (≥8). Students were classified in regard to their KIDMED classification at baseline and follow-up as follows: worsen (from high to poor/moderate or moderate to poor), poor–stable (poor adherence in baseline and follow-up), MH–stable (MH adherence in baseline and follow-up), or improved (from poor to MH or moderate to high). For each quality-of-life score (HRQoL, physical function, emotional function, social function, school function), a score difference was calculated (score at follow-up − score at baseline) and then recoded into a two-group categorical variable indicating improvement (values > 0) or non-improvement (values ≤ 0) in quality of life. Data are presented as mean (standard deviation) for normally distributed continuous variables (age) and % of the corresponding sample for categorical variables. For normally distributed variables (age), *p*-values were obtained using one-way analysis of variance and the Bonferroni correction in the case of post hoc analysis. For categorical variables, a chi-squared test was performed. Abbreviations: International Obesity Task Force (IOTF); Lambda Mu and Sigma (LMS); Standard Deviation (SD); Body Mass Index (BMI); Moderate/High (MH); Health-Related Quality of Life (HRQoL).
